# Development of a measurement approach to assess time children participate in organized sport, active travel, outdoor active play, and curriculum-based physical activity

**DOI:** 10.1186/s12889-018-5268-1

**Published:** 2018-03-22

**Authors:** Michael M. Borghese, Ian Janssen

**Affiliations:** School of Kinesiology and Health Studies, Queen’s University, 28 Division St, Kingston, ON K7L 3N6 Canada

**Keywords:** Movement behaviors, Spatial behavior, Play, Youth sport, Physical education, Active transportation

## Abstract

**Background:**

Children participate in four main types of physical activity: organized sport, active travel, outdoor active play, and curriculum-based physical activity. The objective of this study was to develop a valid approach that can be used to concurrently measure time spent in each of these types of physical activity.

**Methods:**

Two samples (sample 1: *n* = 50; sample 2: *n* = 83) of children aged 10–13 wore an accelerometer and a GPS watch continuously over 7 days. They also completed a log where they recorded the start and end times of organized sport sessions. Sample 1 also completed an outdoor time log where they recorded the times they went outdoors and a description of the outdoor activity. Sample 2 also completed a curriculum log where they recorded times they participated in physical activity (e.g., physical education) during class time.

**Results:**

We describe the development of a measurement approach that can be used to concurrently assess the time children spend participating in specific types of physical activity. The approach uses a combination of data from accelerometers, GPS, and activity logs and relies on merging and then processing these data using several manual (e.g., data checks and cleaning) and automated (e.g., algorithms) procedures. In the new measurement approach time spent in organized sport is estimated using the activity log. Time spent in active travel is estimated using an existing algorithm that uses GPS data. Time spent in outdoor active play is estimated using an algorithm (with a sensitivity and specificity of 85%) that was developed using data collected in sample 1 and which uses all of the data sources. Time spent in curriculum-based physical activity is estimated using an algorithm (with a sensitivity of 78% and specificity of 92%) that was developed using data collected in sample 2 and which uses accelerometer data collected during class time. There was evidence of excellent intra- and inter-rater reliability of the estimates for all of these types of physical activity when the manual steps were duplicated.

**Conclusions:**

This novel measurement approach can be used to estimate the time that children participate in different types of physical activity.

**Electronic supplementary material:**

The online version of this article (10.1186/s12889-018-5268-1) contains supplementary material, which is available to authorized users.

## Background

Physical activity is an important health determinant in children [1]. The four main types of physical activity that children engage in are organized sport (e.g., soccer game, gymnastics practice), active travel (e.g., walking to school), outdoor active play (e.g., exploring nature, playing at recess), and curriculum-based physical activity (e.g., physical education) [2, 3]. It is important to consider time spent in these different types of physical activity because they may have unique determinants [4–9] and health consequences [10]. Moreover, the type of physical activity should be considered when examining physical activity levels and trends in the population because doing so provides insights into the types of physical activity that need to be addressed in order to increase overall physical activity.

Valid methods are available to measure the daily (or weekly) total time spent in some types of physical activity, such as organized sport [11] and active travel [12], but not others, most notably outdoor active play. Furthermore, an approach for concurrently measuring time spent in these different types of physical activity has not been established. Accordingly, the objective of this paper was to describe the development of a measurement approach that can be used to concurrently assess the time children spend participating in organized sport, active travel, outdoor active play, and curriculum-based physical activity.

## Methods

### Background

Our thought processes in developing the measurement approach were driven by the following definitions, premises, and assumptions:

- Organized sport refers to physical activities that are scheduled, typically governed by rules and regulations, often competitive in nature, and supervised and managed by adults. Active travel refers to human-powered transportation such as walking or bicycling [13]. Outdoor active play refers to unstructured and informal physical activities that are freely chosen, typically child-led with little or no adult supervision, and which occur in a variety of outdoor locations [2]. Curriculum-based physical activity refers to physical activity performed during class-time at school and includes physical education classes, special school events that are movement oriented, and in our home province of Ontario, Daily Physical Activity (i.e., 20 min/day of physical activity performed in class). The different types of physical activity were considered mutually exclusive and children could only be classified as partaking in one type of physical activity at a time.
- We aimed to develop a measurement approach that relied upon devices and instruments already used by physical activity researchers. Because objective devices are more valid than self-reported instruments, whenever possible objective devices were chosen. We required that these devices be comfortable to wear and not hinder normal physical activities. Our decisions were guided by the fact that objective devices meeting these criteria are available to measure the intensity of movement (i.e., accelerometer) and the geographic location where movement occurs (i.e., GPS watch).
- We only considered including a self-reported instrument in the final measurement approach if it could be completed quickly by children as young as age 10 in a valid manner. To that end, we felt that children could quickly and reliably record organized sport on an activity log because organized sport sessions start and end at prescheduled times that are often consistent week-to-week. Indeed, the test-retest reliability of recalled organized sport participation is excellent, with intraclass correlation coefficients of 0.95 [11].
- We believed that children would not be able to quickly and reliably recall times spent in active travel, outdoor active play, and curriculum-based physical activity because, in many instances, these activities are not prescheduled, they start and end at varying times and last for different durations, and they are very intermittent. We decided a priori that the measurement approach would need to use algorithms to predict when children engaged in these activities using data from objective devices. For active travel, we used data from a GPS logger and an established software package and algorithm that have previously been shown to provide valid estimates [12]. For outdoor active play and curriculum-based physical activity we aimed to develop new algorithms that would estimate when children were engaged in these activities using data from an accelerometer, GPS logger, and school schedules.

### Study sample

Two samples of children aged 10–13 years were recruited from Kingston, Ontario, Canada. Both samples were drawn from a larger study of 458. The first sample consisted of 50 children and it was used to establish and demonstrate the approaches used to assess organized sport, active travel, and outdoor active play. The second sample consisted of 83 children and it was used to establish and demonstrate the approach used to assess curriculum-based physical activity. Ultimately, the two approaches were combined into a single measurement approach that could be used to assess time spent in all four types of physical activity.

Decisions around the sample sizes were governed by our desire to ensure that we studied a heterogeneous sample (mixture of boys and girls, children of different ages, children from different neighborhoods) who had a variety of outdoor active play and curriculum-based physical activity experiences. We aimed to classify children’s physical activity at the 15-s accelerometer epoch-level; thus, with 7-days of data on 50 (sample 1) and 83 (sample 2) children, we anticipated having tens of thousands of epochs of data, which was more than ample for algorithm development purposes.

### Data collection

**Fig. 1:**
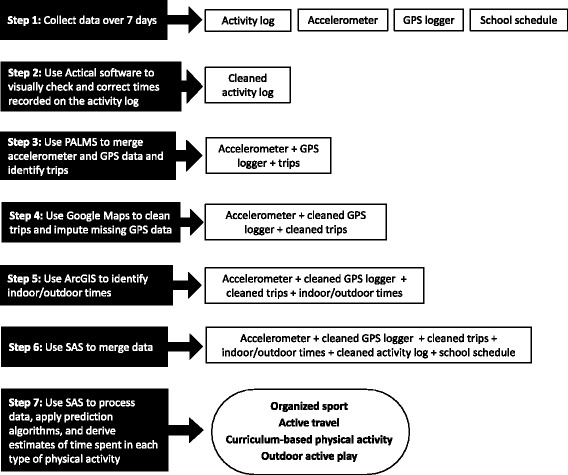
Bland-Altman plots of the average difference between observers for different types of physical activity

Physical activity was measured during waking hours over 7 consecutive days. During these 7 days both samples wore an Actical accelerometer and Garmin Forerunner 220 GPS watch and completed activity logs. Information about participants’ school schedules and calendars were captured in a questionnaire and on school websites. A description of these measurement devices and instruments is provided below. A graphical schematic of the measurement approach developed in this paper can be found in Fig. 1.

#### Accelerometry

Participants were instructed to wear an Actical accelerometer (Philips Respironics, Bend, OR) on their right hip for 24 h/day. The devices were initialized to record data in 15-s epochs starting at midnight on the first measurement day. Participants were instructed to remove the device only for aquatic activities (e.g., swimming, showering).

#### GPS logger

The Garmin Forerunner 220 GPS watch (Garmin, Olathe, KS) recorded the participants’ longitude and latitude positions approximately every 2 s to 2 min depending on satellite signal availability. Geographic positions recorded from this device have a usual accuracy of 5–10 m [14]. The GPS logger has a rechargeable battery that allows for the collection of 11 h of data. Participants charged the GPS logger briefly in the late afternoon or early evening, and overnight while they were sleeping.

#### Activity logs

All participants completed a daily activity log where they recorded their bed and wake times, times that either the accelerometer or GPS watch were not worn, and the start and end times of organized sport and outdoor chores.

The first sample of 50 children completed an additional log, which is hereafter referred to as the outdoor time log, where they recorded times they went outdoors and their main activity when outdoors in 15-min increments. Note that the outdoor time log is not part of the final measurement approach. Rather, information from this log was used to develop the outdoor active play prediction algorithm.

The second sample of 83 children also completed an additional log, which is hereafter referred to as the curriculum-based activity log, wherein they recorded the start and end time of physical activity sessions that occurred during curriculum time including physical education classes and Daily Physical Activity. Note that the curriculum-based activity log is not part of the final measurement approach. Rather, information from this log was used to develop the curriculum-based physical activity prediction algorithm.

#### School schedules

With the help of a parent, participants responded to a questionnaire that asked about the start and end times of their school day, and the start and end times of their recess periods. School and non-school days were determined by checking school websites. Children participating during the spring break (mid-March) and summer holiday (July and August) were asked to indicate the date and times of day camps.

### Data cleaning and processing

#### Cleaning of times recorded in the activity, curriculum-based activity, and outdoor time logs

Actical v3.10 software (Philips Respironics, Bend, OR) was used to visually inspect accelerometer data around times when participants indicated that they were sleeping, not wearing the accelerometer, or engaging in organized sport or outdoor chores. If needed, the start and end times were adjusted to reflect when movement was first detected or stopped being detected by the accelerometer. This approach was repeated for times recorded on the curriculum-based activity log. Times recorded on the outdoor time logs were manually checked by visually examining the GPS positional data using Google Maps (Google, Mountain View, California, USA). If needed, the start and end times were adjusted to reflect the transition between indoor and outdoor environments.

#### Merger of accelerometer and GPS data

After data collection, accelerometer and GPS data were downloaded using the respective proprietary software and were then imported into the Personal Activity and Location Measurement System (PALMS) software [15]. PALMS merged the accelerometer and GPS data according to the accelerometer epochs so that each 15-s epoch was assigned the corresponding GPS longitude and latitude coordinates. PALMS provides a means of merging accelerometer and GPS data using time-stamps, filtering extreme observations and interpolating invalid GPS points caused by brief signal loss [16].

#### Identification of trips and trip modality

PALMS identified trips using a validated algorithm that is based on speed and distance determined from the GPS data [16]. Initially, trips were identified if movement occurred over a distance of ≥100 m, lasted ≥3 min, and if < 90% of the points occurred within a 30 m radius. Trips could be paused if participants stopped for < 3 min. PALMS classified trips as either active (i.e., walking and bicycling at < 25 km/h) or passive (≥25 km/h) according to the 90th percentile of speed during the trip [12].

#### Cleaning of identified trips

The algorithm PALMS used to detect trips was highly sensitive and resulted in the identification of several false positives (e.g., movement occurring on school grounds during recess). The GPS data for each trip identified by PALMS were visually inspected using Google Maps and false positive trips were manually deleted.

#### Cleaning of missing GPS data

Although PALMS interpolated GPS latitude and longitude coordinates for up to 10 min if geographic position was not recorded by the GPS logger [17], some GPS data were still missing. For each time period with missing GPS data Google Maps was used to determine the participant’s location immediately before the GPS signal was lost and immediately after it returned. If there was no change in location, the GPS coordinates for that location were imputed. Additionally, since some of our Geographic Information System (GIS) data were limited to the City of Kingston (see details below), GPS points that were recorded as being outside of Kingston were identified by visually inspecting the GPS data on a map, and then the latitude and longitude coordinates of these points were replaced with GPS coordinates of a similar type of location within Kingston.

#### Identification of indoor and outdoor times using GPS data in GIS

The next step of data processing began by opening the combined accelerometer and GPS data in ArcMap version 10.5 software (ESRI, Redlands, CA). The latitude and longitude coordinates were geocoded as a map layer. A second GIS layer which consisted of building footprints for all buildings within Kingston was opened, and the join function was used to combine the two layers. Each of the epochs were categorized as either indoors or outdoors based on whether its latitude/longitude coordinates fell within a building. We then applied an algorithm to correct for GPS error (see ‘Generation of algorithms’ subsection).

#### Merger of accelerometer, GPS, and GIS data with information from school schedules and from the activity, outdoor time, and curriculum-based activity logs

SAS version 9.4 software was used to merge school schedule/calendar data and the cleaned times from participants’ logs with the file that contained the merged accelerometer, GPS, and GIS data. We then developed a SAS program to determine time spent in the different types of physical activity. The program started by removing all epochs that occurred during sleep or during non-wear time, which included times that children identified as not having worn the accelerometer and the standard procedure whereby 60 consecutive minutes of zero counts, allowing for up to two non-zero minutes, were removed [18]. A valid measurement day was defined as ≥10 h of both accelerometer and GPS data [18]; all epochs from invalid days were deleted. Movement intensity for each epoch was determined using established cut-offs: moderate-to-vigorous ≥375 counts/15 s, light: between < 375 and ≥ 25 counts/15 s, and sedentary: < 25 counts/15 s [19, 20]. Children accumulated very little vigorous intensity movement and time spent in vigorous intensity movement provided little additional predictive power when developing the algorithms described below. Therefore, we combined moderate and vigorous intensity movement.

#### Identification of organized sport

The SAS program flagged all epochs that occurred during organized sport based on the cleaned times recorded on the activity log. Flagged epochs were summed and the daily average was determined.

#### Identification of active travel

All epochs identified as being part of active travel by PALMS and not removed during the cleaning procedures were summed and the daily average was determined.

#### Identification of outdoor active play

The identification of outdoor active play started by flagging 15-s epochs that could not have occurred during outdoor active play because they occurred during one or more of the following: time in bed, indoors, school curriculum time (but not recess time) or during a day camp in the summer, during active or passive travel, during an organized sport, or performing outdoor chores. For epochs not flagged in the above step, we used the outdoor active play prediction algorithm to estimate whether they occurred during outdoor active play. A detailed explanation of this algorithm and the process used to develop it is provided below in the ‘Generation of algorithms’ subsection. All epochs identified as being part of outdoor active play by this algorithm were summed and the daily average was determined.

#### Identification of curriculum-based physical activity

vThe identification of curriculum-based physical activity started by flagging 15-s epochs that occurred during curriculum time based on information from school schedules and calendars. We then used the curriculum-based physical activity prediction algorithm to estimate whether or not these epochs occurred during curriculum-based physical activity. This algorithm is explained in the ‘Generation of algorithms’ subsection. All epochs identified as being part of curriculum-based physical activity by this algorithm were summed and the daily average was determined.

### Generation of algorithms

#### Indoor/outdoor algorithm

When visually inspecting the GPS data we noticed that many of the coordinates were outdoors but within a few meters of a building. Oftentimes, the time-stamps of these coordinates indicated that they were immediately preceded and followed by coordinates that were indoors. We also noticed that some of the coordinates that were located indoors were immediately preceded and followed by coordinates that were outdoors but within a few meters of the building. These two scenarios reflect measurement error of the GPS logger. We created an algorithm that corrected this error by reclassifying coordinates such as those explained in the two scenarios above. The algorithm was developed using data from the 50 participants who completed the outdoor time log. The outdoor times recorded on these logs were used as the reference during algorithm development. Parameters for the algorithm were selected with the intent of providing a sensitivity of 95% for predicting outdoor time while maximizing specificity. When developing the algorithm, each 15-s epoch was coded as either a 1, if outdoors, or a 0, if indoors based on the GPS and GIS data. Then, for each 15-s epoch, a 10-min and 20-min forward-rolling average was created based on these 1 and 0 values. We then determined which forward-rolling average variable and cut-off best predicted outdoor time based on the log recordings. A cut-off of 62.5% for the 10-min forward-rolling average provided a sensitivity of 95% while maximizing specificity, which in this case was 76%. Therefore, epochs with a 10 min-rolling average of ≥62.5% were classified as outdoors and values < 62.5% were classified as indoors.

#### Outdoor active play algorithm

The final measurement approach uses an algorithm to predict outdoor active play during periods when children were outdoors but not sleeping, or during school hours (other than recess) or day camps, or engaged in active or passive travel, organized sport, or chores. The algorithm was developed using data from the 50 participants who completed the outdoor time log. The times on the logs that reflected sessions of outdoor active play and outdoor sedentary behaviors (e.g., eating outside, reading outside) were used as the reference during the algorithm development, and this reference data was predicted using data from the accelerometer. As noted above, children recorded their outdoor times in 15-min intervals throughout the day. As such, these times needed to be adjusted to increase precision (i.e., to the closest minute of going outdoors or returning indoors). Google Maps was used to inspect GPS data around these times and times were adjusted if necessary. A total of 127 sessions of outdoor active play were recorded and they contained 16,790 epochs. A total of 920 sessions of outdoor sedentary behavior were recorded and they contained 137,590 epochs. During the algorithm development the outcome variable for each 15-s epoch was coded as a 1 if the participant recorded on the outdoor time log that they were engaged in outdoor active play, or a 0 if they recorded that they were engaged in a sedentary behavior. We then created 31 independent variables for each epoch. The first 3 independent variables were continuous variables that reflected the proportion of time during the outdoor session that was spent at a sedentary, light, and moderate-to-vigorous intensity. The next 21 independent variables were dichotomous variables that reflected whether or not the epoch was contained within a 10-min bout where at least 3, 4, 5, 6, 7, 8, or 9 min was in the sedentary, light, or moderate-to-vigorous intensity range. The next 6 independent variables were continuous variables that reflected the 5-min, 10-min, and 20-min centered- and forward-rolling averages of the 15-s epoch count values. The final independent variable reflected the duration of the outdoor session in which the epoch was contained. We then determined, in an exploratory manner, which combinations of these 31 independent variables could be used to best distinguish between outdoor active play and outdoor time spent in sedentary behavior. All of the decision rules and parameters selected for the algorithm were made with the intent of maximizing the combined sensitivity and specificity of predicting outdoor active play.

Ultimately, the following criteria were defined during the algorithm development process: 1) the epoch was within an outdoor session where ≥57% of time was in the sedentary intensity range, 2) the epoch was within an outdoor session where ≤3% of time was in the moderate-to-vigorous intensity range, 3) the epoch was within an outdoor session where > 52% of time was in the sedentary intensity range, 4) the epoch was within a bout where at least 7 of 10 min was in the sedentary intensity range, 5) the epoch was within a bout where at least 8 of 10 min was in the sedentary intensity range, 6) the centered 5-min rolling average of the epoch was < 180 counts/15 s, 7) the 20-min forward rolling average was < 90 counts/15 s, and 8) the 20-min centered-rolling average was < 334 counts/15 s. Based on these criteria, an epoch was classified as being part of session of outdoor sedentary behavior if *any* of the following combinations of criteria were met: (criteria 1 and 2), or (criteria 3 and 4), or (criteria 5 and 6), or (criteria 5, 7, and 8). Epochs that did not meet any of these combinations of criteria were classified as being part of outdoor active play as long as they did not occur during periods of time when children were indoors, sleeping, during school hours (other than recess), or engaged in active or passive travel, organized sport, or chores.

#### Curriculum-based physical activity algorithm

The final measurement approach uses an algorithm to predict curriculum-based physical activity during school curriculum time. This algorithm was developed using data from the sample of 83 children who recorded the times of their physical education and Daily Physical Activity sessions on the curriculum time log. The times on the logs that reflected sessions of curriculum-based physical activity and sessions of passive curriculum time (e.g., times when participants did not record being engaged in curriculum-based physical activity) were used as the reference during the algorithm development process, and this reference data was predicted using data from the accelerometer. A total of 183 sessions of curriculum-based physical activity were recorded and they contained 30,077 epochs. A total of 1268 sessions of passive curriculum time were recorded and they contained 511,060 epochs. During the algorithm development the outcome variable for each 15-s epoch was coded as a 1 if the participant recorded that they were engaged in curriculum-based physical activity or a 0 if the participant did not record any curriculum-based physical activity during this time. We then created the same 31 independent variables for each epoch that were created when developing the outdoor active play algorithm. We then determined, in an exploratory manner, which combinations of these 31 independent variables could be used to distinguish between curriculum-based physical activity and passive curriculum time. All of the decision rules and parameters selected for the algorithm were made with the intent of maximizing the sensitivity and specificity of predicting curriculum-based physical activity.

The following criteria were defined during the algorithm development process: 1) the epoch was within a session of curriculum time where < 5% of time was in the moderate-to-vigorous intensity range, 2) the epoch was within a session of curriculum time where ≥33% of time was in the sedentary intensity range, 3) the epoch was within a session of curriculum time that had a duration > 15 min and < 100 min, 4) the epoch was within a bout where less than 5 of 10 min was in the light intensity range, 5) the epoch was within a bout where at least 7 of 10 min was in the sedentary intensity range, 6) the centered 20-min rolling average of the epoch was <377 counts/15 s. Based on these criteria, an epoch was classified as being part of session of passive curriculum time if *any* of the following combinations of criteria were met: (criteria 1 and 2), or (criteria 3), or (criteria 4, 5, and 6). Epochs that occurred during curriculum time and did not meet any one of these combinations of criteria were classified as being part of curriculum-based physical activity.

### Statistical analysis

Statistical analyses were done using SAS 9.4. Statistical significance was set at *p* < .05. Descriptive values are presented as mean (SD) unless otherwise indicated. A brief overview of the SAS syntax required to implement the measurement approach described in this paper can be found in Additional file 1.

When developing the outdoor active play and curriculum-based physical activity prediction algorithms, we started by generating multiple algorithms that reflected how the different independent variables, their combinations, and their interactions predicted the reference data. From these many possibilities we selected the algorithm that maximized the combined sensitivity and specificity.

**Fig. 2:**
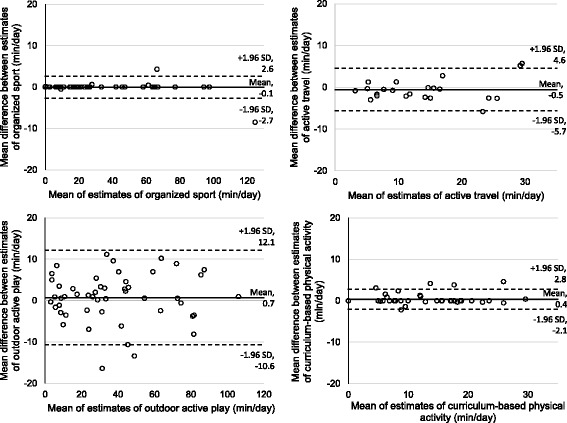
Schematic of data collection and development of the measurement approach for assessing the time spent in outdoor active play, organized sport, active travel, and curriculum-based physical activity

Some of the steps for deriving physical activity estimates involved cleaning procedures that are subjective. Therefore, all manual cleaning procedures were completed in triplicate – twice by the same observer and a third time by a different observer – to establish intra- and inter-rater reliability. Two-way random, single measure intra-class correlation (ICC (2,1); denoted as ICC) and one-way ANOVA were used to describe the relationship and differences between estimates derived for intra- and inter-rater reliability, respectively. Bland-Altman plots of the average difference between observers can be found in Fig. 2.

## Results

### Descriptive characteristics and compliance

In the sample of 50 that were used to develop the organized sport, active travel, and outdoor active play portions of the measurement protocol the mean combined accelerometer and GPS wear time was 14.8 (1.6) hours/day and data were available from 328 days. The mean age was 11.4 (1.1) years and 50% were male. In the sample of 83 that were used to develop the curriculum-based physical activity portion of the measurement protocol, the mean combined accelerometer and GPS wear time was 13.3 (0.8) hours/day and data were available from 491 days. The mean age was 11.9 (1.0) years and 51% were male. In sample 1, 34/50 had normal weight, 9/50 had overweight and 7/50 had obesity. In sample 2, 64/83 had normal weight, 12/83 had overweight, and 7/83 had obesity. Average minutes/day of moderate-to-vigorous physical activity, light physical activity, and sedentary behavior for both samples is in Table 1.

**Table 1:** Description of movement intensity in two samples of children

Time spent in movement behavior	Sample 1 (*n* = 50)	Sample 2 (*n* = 83)
Moderate-to-vigorous physical activity	54.5 (47.5, 61.5)	61.3 (55.7, 66.8)
Light physical activity	156.6 (147.3, 165.8)	157.7 (151.7, 163.6)
Sedentary time	674.2 (647.2, 701.1)	577.4 (564.3, 590.6)

Values are presented as mean (95% CI)

### Organized sport

There was evidence of excellent intra- and inter-rater reliability for the organized sport estimates after all of the manual cleaning steps were repeated in triplicate – twice by the same observer and a third time by a second observer. The means were within 4.0 min/day (11.5%) and they were not significantly different from each other (*p* = .99) (Table 2). The three values were highly correlated (ICC = .99, *p* < .00001). Bland-Altman plots revealed excellent agreement with no evidence of systematic error (Fig. 2).

**Table 2:** Intra- and inter-rater reliability of children’s time (minutes/day) spent in four types of physical activity

Type of physical activity	Observer 1, round 1	Observer 1, round 2	Observer 2	ICC
Organized sport	34.9 (24.2, 50.3)	34.9 (24.3, 50.3)	30.9 (22.6, 42.1)	0.99
Active travel	14.8 (12.3, 17.3)	15.9 (13.5, 18.3)	14.7 (12.7, 16.8)	0.95
Curriculum-based physical activity	10.3 (8.7, 12.2)	9.9 (8.3, 11.8)	9.8 (8.2, 11.7)	0.98
Outdoor active play	41.0 (31.6, 53,2)	46.2 (34.0, 62.6)	40.8 (30.5, 54.6)	0.96

Values are presented as mean (95% CI) derived using a two-part model

*ICC* two-way random, single measure (2,1) intra-class correlation

### Active travel

There was evidence of excellent intra- and inter-rater reliability for the active travel estimates after all of the manual cleaning steps were repeated in triplicate. The means were within 1.2 min/day (7.5%) and were not significantly different from each other (*p* = .71) (Table 2). The three values were highly correlated (ICC = .95, *p* < .00001). Bland-Altman plots revealed excellent agreement with no evidence of systematic error (Fig. 2).

### Outdoor active play

There was evidence of excellent intra- and inter-rater reliability for adjusting the outdoor times reported on the log. Approximately 99.2% (intra-rater) and 97.4% (inter-rater) of the adjusted outdoor times were < 5 min based on repeated attempts. The algorithm developed to predict outdoor active play at the epoch-level did so with both a sensitivity and specificity of 85% when compared with children’s reported outdoor active play as the reference. There was evidence of excellent intra- and inter-rater reliability for the outdoor active play estimates after all of the manual cleaning steps were repeated in triplicate. As seen in Table 2, the means were within 5.4 min/day (11.7%) and were not significantly different from each other (*p* = .80). The three values were highly correlated (ICC = .96, *p* < .00001). Bland-Altman plots revealed acceptable agreement with no evidence of systematic error (Fig. 2).

### Curriculum-based physical activity

At the epoch-level the algorithm developed to determine curriculum-based physical activity correctly distinguished between physical activity-related curriculum time and passive curriculum time with a sensitivity and specificity of 78% and 92%, respectively. There was evidence of excellent intra- and inter-rater reliability for the curriculum-based physical activity estimates after all of the manual cleaning steps were repeated in triplicate. The means were within 0.5 min/day (4.8%) and were not significantly different from each other (*p* = .97) (Table 2). The three values were highly correlated (ICC = .98, *p* < .00001). Bland-Altman plots revealed excellent agreement with no evidence of systematic error (Fig. 2).

## Discussion

This paper describes the development of a methodological approach that can be used to concurrently measure the time children participate in organized sport, active travel, outdoor active play, and curriculum-based physical activity. The final approach provides valid measurements of all these different types of physical activity. It uses a combination of data from an accelerometer, GPS logger, GIS data on indoor/outdoor locations, an activity log where start and end times of organized sport and outdoor chores are recorded, and school schedules. The measurement approach is explained in detail within the Methods section, starting with the “*Data collection*” subsection and ending with the “*Identification of curriculum-based physical activity”* subsection, but excludes all content related to the collection and cleaning of data for the outdoor time log and curriculum time activity log.

In our experience, the measurement approach was positively received and not onerous for the child participants. They informed us that the accelerometer and GPS watch were comfortable to wear and that the accelerometer was small enough to be concealed if desired. This was reflected in the excellent compliance for wearing both the accelerometer and GPS watch. Children and parents also indicated to us that the activity log could be completed quickly and easily, and in the majority of instances we did not need to make corrections to the times recorded on the daily activity and curriculum-based physical activity logs during the data cleaning process. Times recorded on the outdoor time log, by design, frequently had to be adjusted to increase precision (i.e., to the closest minute of going outdoors or returning indoors) because they were only recorded in 15-min intervals.

The newly developed measurement approach involves several manual cleaning procedures as well as several automated steps for merging and processing the data. Collectively, these steps are very time- and labor-intensive and they took our research team approximately 1 working day per participant to complete. While many of these cleaning and processing steps are essential, some may not be. Specifically, researchers may choose to not examine recoded times for organized sport (very few adjustments were made in our study), non-wear time (researchers could use standard procedures alone to identify non-wear time [18]), or sleep (participants could be asked to remove the device for sleep or algorithms could be used to predict sleep times using accelerometer data [21]). Researchers could also use a GPS logger that uses satellite signal-to-noise ratios to distinguish outdoor and indoor time, rather than using a purpose-built algorithm, because the accuracy of these two methods is similar [22]. In addition, studies using a school-based sampling approach could use school timetables to estimate time spent in curriculum-based physical activity rather than an algorithm. Some aspects of the measurement approach developed in this study, such as the outdoor active play and curriculum-based physical activity algorithms, would need to be adapted if different accelerometer devices, epoch lengths, or populations were being studied. Additionally, we assumed that the different types of physical activity are mutually exclusive; however, this may not always be the case. For example, children could engage in outdoor play as part of their walk home from school. Moreover, there are other types of physical activity that children engage in (e.g., indoor active play) that were not captured in this study.

This approach could be applied in observational studies to estimate the amount of time that children spend engaging in various types of physical activity. Alternatively, this approach could be applied in intervention settings to evaluate the effectiveness of interventions that target a specific type (or types) of physical activity and to determine if changes in participation in one type of physical activity displace time spent in another. However, the large quantity of data and the high demand for computing and human resources make this approach infeasible for population-level surveillance and monitoring.

To our knowledge, this is the first study to measure outdoor active play under free-living conditions over the course of a full week. The approach we developed uses an algorithm to predict when children are engaged in outdoor active play using a combination of data from many sources. This algorithm was developed using an outdoor time log to capture reference data for time that children spent in outdoor active play. This log is similar to a time-use log, which is thought to be more accurate than other self-report measures of behavior [23–25]. In this way, we used physical activity intensity data during times that children identified as playing outdoors to develop a predictive algorithm, rather than imposing our own impression of how children should be moving during outdoor active play.

Previous studies that have combined accelerometry and GPS data from children to measure physical activity have classified physical activity according to geographic location (e.g., near home, in a park) [17, 26–29]. While informative, location alone cannot be used to determine what type of physical activity the child was engaged in. For instance, physical activity accumulated in a park could reflect any of the four types of physical activities measured in the current study.

## Conclusion

This paper describes the development of a methodological approach for concurrently measuring different types of physical activity that children engage in. We hope that this approach will provide researchers with a new opportunity to better measure and study the time spent in different types of physical activity.

## Additional file

Additional file 1: SAS syntax for deriving estimates of time that children spend in different types of physical activity. (PDF 174 kb)

## Abbreviations

GIS: Geographic Information System
GPS: Global Positioning System
ICC: Intraclass correlation
PALMS: Personal Activity and Location Measurement System
