# Supplementary material for: Development of a measurement approach to assess time children participate in organized sport, active travel, outdoor active play, and curriculum-based physical activity
Source: BMC Public Health. 2018 Mar 22;18:396. doi: 10.1186/s12889-018-5268-1 (PMC5865369; doi:10.1186/s12889-018-5268-1)
Supplement: Supplementary file 1 — SAS syntax for deriving estimates of time that children spend in different types of physical activity. (PDF 174 kb) [file 12889_2018_5268_MOESM1_ESM.pdf]

```
******************************************************************************
ADDITIONAL FILE 1 -  Development of a measurement approach to assess time
children participate in outdoor active play, organized sport, active travel,
and curriculum-based physical activity
Borghese MM, Janssen I
BMC Pubic Health


SAS SYNTAX FOR DERIVING ESTIMATES OF TIME THAT CHILDREN SPEND IN DIFFERENT
TYPES OF PHYSICAL ACTIVITY


This code is meant to serve as a starting point for researchers to replicate
two algorithms that we describe in the above paper - one for deriving
estimates of
outdoor active play and the other for deriving estimates of curriculum-based
physical activity. To use this code you will need to have an intermediate
understanding of SAS syntax and be familiar with methods that are commonly
used when collecting, managing, and analyzing accelerometer and GPS data in
children.

For a visual schematic of data management and reduction, refer to Figure 1 in
the paper.
*****************************************************************************



*****************************************************************************
1 - Overview of data collection

Accelerometer and GPS data will need to be collected, and information on
participants' organized sport times, sleep times, and school schedules is
required.
*****************************************************************************


*****************************************************************************
2 - Preliminary data processing

There are several preliminary data processing steps that are required in
order to derive the final dataset.

2.1 Following data collection, accelerometer and GPS must be merged according
      to accelerometer epoch. A variety of software packages could be used to
      do this. We merged these data using the Personal Activity and Location
      Measurement System (PALMS): https://palms.ucsd.edu:8443/PALMS/. PALMS
      is a free service provided by the Center for Wireless and Population
      Health Systems at the University of California, San Diego
      (https://ucsd-palms-project.wikispaces.com/). If you would like to know
      which parameters and values were selected within PALMS for the current
      paper, please contact the authors (Mike Borghese,14mmb4@queensu.ca; Ian
      Janssen, ian.janssen@queensu.ca).

2.2 After the accelerometer and GPS data have been merged (e.g., using PALMS)
      you may wish to visually inspect GPS data, and if possible, impute
      missing GPS data. You may also wish to visually inspect trips
      identified by PALMS and add delete falsely identified trips. These
```

steps are explained in more detail in the paper. Although these steps are not necessary, it our experience they were important.

2.3 Identify time spent indoors and outdoors. Obtain GIS data from the geographic region that you collected accelerometer and GPS data. Combine the cleaned, merged accelerometer and GPS data with building footprint information to determine whether a GPS point is indoors or outdoors using ArcGIS or similar GIS software. You may want to develop your own approach to clean this indoor/outdoor variable for GPS jitter and GPS drift. An alternative approach for this step would be to use a satellite signal-to-noise ratio built into the GPS device to determine if each of the GPS data collection points occurred while the participant was indoors or outdoors.

2.4 Import and concatenate all of these files into SAS. Flag all the relevant times that participants recorded on their activity logs - including time spent sleeping, participating in organized sports and school schedule information (day of the week, start and end times of school and recess).

2.5 Identify accelerometer non-wear time and categorize movement into intensity categories. This will be specific to the device used, wear location, epoch length, and population being studied.

2.6 Derive a dataset containing only those participants with sufficient wear time. In our study we required that participants have at least 4 days with at least 10 hours of paired accelerometer and GPS data (after having removed accelerometer non-wear time and missing GPS data). This is the primary dataset that should be used for the code below.
****

****
3 - Derive estimates of organized sport participation and active travel

In the dataset derived in Step 2.6, output estimates of time spent in organized sport and active travel.
****

****
4 - Derive variables needed for prediction algorithms

At this point, the dataset should contain the following variables:

Identifier - participant ID variable
Sequence - number of accelerometer epochs for each participant, in sequence
Activity – accelerometer activity count for that epoch
Outdoor - denotes whether each epoch occurred while the participant was indoors or outdoors (*note*: in our study 0 referred to indoors and 1 referred to outdoors)
Sleeping - denotes whether each epoch occurred while the participant was sleeping or not sleeping (*note*: in our study 1 referred to sleep periods and 0 referred to non-sleep periods)
Nonwear - denotes whether the accelerometer was worn during this epoch (*note*: in our study 1 referred to epochs recorded during non-wear time and 0 referred to epochs recorded while the accelerometer was worn)

```
GPS_missing - denotes whether the GPS logger was worn during this epoch and
      GPS data were recorded (note: in our study 1 referred to epochs that
      had missing GPS data or during which time the GPS logger was not worn
      while 0 refers to epochs with useable GPS logger data).
School - denotes school hours, i.e., a school day during school hours, but
      excludes recess time during the school day (note: in our study 1
      referred to epochs that occurred during school curriculum-time and 0
      referred to epochs that did not occur during school curriculum time)
Recess - denotes whether the participant was in school recess during this
      time, i.e., a school day during a school recess period (note: in our
      study 1 referred to epochs recorded during recess and 0 referred to
      epochs that did not occur during recess)
Trip - denotes whether the participant was engaged in a trip (active or
      passive) during this time (note: in our study 1 referred to epochs
      recorded during a trip and 0 referred to epochs recorded while not in a
      trip)
Sports - denotes whether the participant was engaged in an organized sport
      during this time (note: in our study 1 referred to epochs recorded
      during an organized sport and 0 referred to epochs recorded while not
      participating in an organized sport)

4.1 Create several rolling averages of physical activity intensity for each
      epoch. The code below produces 5-min, 10-min, and 20-min centred- and
      forward-rolling averages. Note that '20', '40' and '80' values were used
      in this code because we used 15s epochs. These values would be different
      for other epoch lengths.
      ;

PROC sort data=data1;
by identifier sequence;
run;
proc expand DATA = data1 OUT = data2;
      by identifier;
      id sequence;
      convert activity = mAVGcount_F20 / METHOD = none TRANSFORMOUT =
            (reverse movave 20 reverse);
      convert activity = mAVGcount_C20 / METHOD = none TRANSFORMOUT =
            (cmovave 20);
      convert activity = mAVGcount_F40 / METHOD = none TRANSFORMOUT =
            (reverse movave 40 reverse);
      convert activity = mAVGcount_C40 / METHOD = none TRANSFORMOUT =
            (cmovave 40);
      convert activity = mAVGcount_F80 / METHOD = none TRANSFORMOUT =
            (reverse movave 80 reverse);
      convert activity = mAVGcount_C80 / METHOD = none TRANSFORMOUT =
            (cmovave 80);
      label       mAVGcount_F20=mAVGcount_F20   mAVGcount_C20=mAVGcount_C20
                  mAVGcount_F40=mAVGcount_F40   mAVGcount_C40=mAVGcount_C40
                  mAVGcount_F80=mAVGcount_F80   mAVGcount_C80=mAVGcount_C80;
run;

      *
4.2 Identify movement intensity bouts (i.e., 2 - 9 /10 minutes for sedentary,
      light, moderate, vigorous, and moderate-to-vigorous intensities). This
      can be done using step #3 of the code developed by Boudreau & Belanger
      (http://mathieubelanger.recherche.usherbrooke.ca/Actical.htm#2-5.) for
```

```
      data collected in epoch lengths shorter than or equal to 1 minute.
      Alternatively, the ACCEL+ code developed by Rachel Colley and the HALO
      group can be used for data collected in 1 minute epochs
      (http://www.haloresearch.ca/accel/).

Once the start and end times of each bout length is identified, these times
can be combined with the primary dataset containing merged accelerometer,
GPS, activity log, and indoor/outdoor data. An example of a macro to do this
is provided below
      ;

%macro merge;     *Change all four of these to _'intensity'XofY - i.e., for
                  bouts of MVPA lasting 8 of 10 minutes, write: _mv8of10;
%let i = _mv8of10;
%let j = _mv8of10_start;
%let k = _mv8of10_end;
%let l = _mv8of10_wide;

data output.Out&i;
      set output.Out&i;
      identifier=input(am_identify_no, 4.);
      if end_mv=0 then delete;
      drop clinicid am_identify_no;
run;
PROC sort data=output.Out&i;
      by identifier dayworn mv_num;
run;
data output.Out&i;
set output.Out&i;
      by identifier dayworn mv_num notsorted;
      retain group;
      if first.identifier then group=1;
      else group+1;
run;
proc transpose data=output.Out&i out=output.Out&j prefix=start;
      by identifier;
      id group;
      var strt_mv;
run;
proc transpose data=output.Out&i out=output.Out&k prefix=end;
      by identifier;
      id group;
      var end_mv;
run;
data output.Out&l;
      merge output.Out&j output.Out&k;
      by identifier;
      drop _name_;
run;
%mend;
%merge;

%macro flagmv8;   *Adjust q for the number of bouts;
%let i = _mv8of10;
%let l = _mv8of10_wide;
%let q=35;
data data2;
```

```
merge data2 output.out&l;
      by identifier;
run;
data data2;
set data2;
      bout&i=0;
      array start (*) start1--start&q;
      array end (*) end1--end&q;
            do i=1 to &q;
                  if start(i) <=    sequence1   <=    end(i)      then
bout&i=1;
            end;
run;
data data2;
set data2;
      drop
      start1 -- end&q i;
run;
%mend;
%flagmv8;
*****************************************************************************


*****************************************************************************
5 - Develop and apply outdoor active play algorithm

The algorithm provided below is meant as a starting point for researchers to
develop their own unique approach to predicting time spent in outdoor active
play. It is provided as a guide, and by no means will it provide a valid
estimate of time spent in outdoor active play in other studies/samples.

5.1 Create a dataset containing only time that could possibly be considered
      outdoor active play. Time spent sleeping, during school, or
      participating in organized sports or active travel are removed. Time
      when children did not wear either device is also removed. Also create
      an initial prediction variable. Note that you could do this step prior
      to step 2.6, which would improve efficiency.

*Set-up an initial prediction variable
OAP_pred - initial prediction variable. Is equal to 0 if outdoor active play
      is not likely to have occurred at this time and is set to 99 if outdoor
      active play is probable during this time.
bout_'intensityXofY' - Denotes whether an individual epoch is contained with
      a bout of movement intensity. For example, "bout_sed8of10=1" means that
      this epoch is contained with a bout of at least 8 out of 10 minutes of
      sedentary time.
ACCELnonwear - nonwear time. Is equal to 1 if device is not being worn at
      that time, or 0 if it is being worn.
GPS_miss - GPS nonwear time. Is equal to 1 if device is not being worn at
      that time, or 0 if it is being worn.
in_school - denotes whether a participant is in school (1) or not (0)
trip/daycamp/sports - 3 variables which denote whether a participant was
      engaged in this pursuit at this time (1) or not (0)
Outdoor - denotes whether a participant is outdoors (1) or not (0)
      ;
data data2_oaponly;
set data2;
```

```
OAP_pred=99;
if bout_sed8of10=1 then OAP_pred =0;
if bout_sed8of10=1 AND mAVGcount_F80>100 then OAP_pred =99;
if sleeping=1 then delete;
if ACCELnonwear=1 then delete;
if gps_miss=1 then delete;
if in_school=1 then delete;
if trip=1 then delete;
if sports=1 then delete;
run;

      *
5.2 Create an outdoor session variable and compute the proportion of time
      spent in each movement intensity during each session. Merge these
      proportions with the primary dataset.
      ;
data data3; *Flag each unique session;
set data2_oaponly;
by identifier day notsorted OAP_pred;
retain group;
      if first.identifier then group=0;
            if first.OAP_pred and OAP_pred=99 then group+1;
            outdoor_session=group;
                  if OAP_pred=0 then outdoor_session=0;
drop group;
run;

PROC summary data=data3; *Output sessions;
by identifier outdoor_session;
      var mvpa vpa mpa lpa sed;
      output out=outdoor_session sum=n_epochs_mvpa n_epochs_vpa n_epochs_mpa
      n_epochs_lpa n_epochs_sed;
where outdoor_session ne 0;
run;

Data outdoor_session;
set outdoor_session;
duration_min=_FREQ_/4;
percent_mvpa=n_epochs_mvpa/_FREQ_;
percent_vpa=n_epochs_vpa/_FREQ_;
percent_mpa=n_epochs_mpa/_FREQ_;
percent_lpa=n_epochs_lpa/_FREQ_;
percent_sed=n_epochs_sed/_FREQ_;
run;
*Merge outdoor_session with data3;
proc sort data=outdoor_session;
by identifier outdoor_session;
run;
proc sort data=data3;
by identifier outdoor_session;
run;
data data3;
merge data3 outdoor_session;
by identifier outdoor_session;
run;
      *
```

```
5.3 Apply the outdoor active play algorithm. There will need to be many
      iterative attempts to develop the best possible algorithm. Below is the
      best possible algorithm that we developed in our study.
      ;

data data3;
set data3;
OAP_pred=99;
      if bout_sed8of10=1 then OAP_pred=0;
      if bout_sed8of10=1 AND mAVGcount_F80>90 then OAP_pred=99;
      if OAP_pred=0 AND mAVGcount_C20 > 180 then OAP_pred=99;
OAP_pred2=OAP_pred;
      if OAP_pred=99 AND percent_sed>0.57 then OAP_pred2=0;
      if OAP_pred=99 AND percent_mvpa<0.03 then OAP_pred2=0;
      if OAP_pred2=0 AND mAVGcount_C80>334 then OAP_pred2=99;
OAP_pred3=OAP_pred2;
      if OAP_pred2=99 AND bout_sed7of10=1 AND percent_sed>0.52 then
      OAP_pred3=0;
run;

      *
5.4 Output estimates of time spent in outdoor active play.
****************************************************************************;



****************************************************************************
6 - Develop and apply curriculum-based physical activity algorithm

An explanation of how to derive many of the variables that are needed for
this algorithm is provided above.

The algorithm provided below is meant as a starting point for researchers to
develop their own unique approach to predicting time spent in curriculum-
based physical activity. It is provided as a guide, and by no means will it
provide a valid estimate of time spent in curriculum-based physical activity
in other studies/samples.

6.1 Start with dataset derived in step 2.6. Remove all data except for epochs
that occur during school hours, but not during recess time (referred to here
as data1_PE). Develop and apply an algorithm to predict time spent in
curriculum-based physical activity. The algorithm developed for our paper is
below.

*Set-up an initial prediction variable
Physed_pred = initial prediction variable. Is equal to 0 if curriculum-based
      physical activity is not likely to have occurred at this time and is
      set to 1 if curriculum-based physical activity is probable during this
      time. Set to 99 by default
            ;
data data1_PE;
set data1_PE;
Physed_pred=99;
      if bout_lt5of10=1 then Physed_pred =1;
      if bout_sed7of10=1 then Physed_pred =0;
      if Physed_pred =0 AND mAVGcount_c80 > 377 then Physed_pred =1;
Physed_pred2=Physed_pred;
```

```
      if Physed_pred=1 AND percent_mvpa_physed_num < 0.05 then
      Physed_pred2=0;
      if Physed_pred=1 AND percent_sed_physed_num > 0.77 then Physed_pred2=0;
run;

      *
6.2 Create curriculum-time session variable and compute the proportion of
      time spent in each movement intensity during each session.
      ;

proc sort data=data1_PE;
by identifier sequence;
run;
data data1_PE;
set data1_PE;
by identifier notsorted Physed_pred2;
retain group;
      if first.identifier then group=0;
            if first.Physed_pred2 AND Physed_pred2=1 then group+1;
      physed_num=group;
      if Physed_pred2=. then physed_num=.;
      drop group;
run;

proc sort data=data1_PE;
by identifier physed_num;
run;
proc summary data=data1_PE;
by identifier physed_num;
var mvpa vpa mpa lpa sed;
output out=physed_num sum=mvpa_sum vpa_sum mpa_sum lpa_sum sed_sum;
run;

      *
6.2   Merge these proportions with the primary dataset. Remove sessions that
      are <15 minutes or >100 minutes. (Note: we chose 15 and 100 minutes
      based on typical lengths of physical education and daily physical
      activity classes in our local schools)
      ;
data physed_num;
set physed_num;
if physed_num=. then delete;
physed_num_duration=_FREQ_/4;
percent_mvpa_physed_num=mvpa_sum/_FREQ_;
percent_vpa_physed_num=vpa_sum/_FREQ_;
percent_mpa_physed_num=mpa_sum/_FREQ_;
percent_lpa_physed_num=lpa_sum/_FREQ_;
percent_sed_physed_num=sed_sum/_FREQ_;
valid_physed_num=1;
if physed_num_duration le 15 then valid_physed_num=0;
if physed_num_duration > 100 then valid_physed_num=0;
drop _TYPE_ -- sed_sum;
run;

proc sort data=physed_num;
by identifier physed_num;
run;
```

```
proc sort data=data1_PE;
by identifier physed_num;
run;
data data1_PE;
      merge data1_PE physed_num;
      by identifier physed_num;
            if valid_physed_num=0 then Physed_pred2=0;
run;

      *
6.3   Output estimates of time spent in curriculum-based physical activity
      ;
*****************************************************************************;
```
